# The impact of pitolisant, an H_3_ receptor antagonist/inverse agonist, on perirhinal cortex activity in individual neuron and neuronal population levels

**DOI:** 10.1038/s41598-022-11032-y

**Published:** 2022-05-12

**Authors:** Kyosuke Hirano, Yoshikazu Morishita, Masabumi Minami, Hiroshi Nomura

**Affiliations:** 1 Endowed Department of Cognitive Function and Pathology, Institute of Brain Science, Nagoya City University Graduate School of Medical Sciences, 1 Kawasumi, Mizuho-cho, Mizuho-ku, Nagoya, 467-8601 Japan; 2 Department of Pharmacology, Graduate School of Pharmaceutical Sciences, Hokkaido University, Sapporo, 060-0812 Japan

**Keywords:** Learning and memory, Neuronal physiology

## Abstract

Histamine is a neurotransmitter that modulates neuronal activity and regulates various brain functions. Histamine H_3_ receptor (H_3_R) antagonists/inverse agonists enhance its release in most brain regions, including the cerebral cortex, which improves learning and memory and exerts an antiepileptic effect. However, the mechanism underlying the effect of H_3_R antagonists/inverse agonists on cortical neuronal activity in vivo remains unclear. Here, we show the mechanism by which pitolisant, an H_3_R antagonist/inverse agonist, influenced perirhinal cortex (PRh) activity in individual neuron and neuronal population levels. We monitored neuronal activity in the PRh of freely moving mice using in vivo Ca^2+^ imaging through a miniaturized one-photon microscope. Pitolisant increased the activity of some PRh neurons while decreasing the activity of others without affecting the mean neuronal activity across neurons. Moreover, it increases neuron pairs with synchronous activity in excitatory-responsive neuronal populations. Furthermore, machine learning analysis revealed that pitolisant altered the neuronal population activity. The changes in the population activity were dependent on the neurons that were excited and inhibited by pitolisant treatment. These findings indicate that pitolisant influences the activity of a subset of PRh neurons by increasing the synchronous activity and modifying the population activity.

## Introduction

Histamine is a neurotransmitter that regulates wakefulness, motivation, energy balance, and learning and memory^1–3^. The activity of histamine neurons and release of histamine are dynamically modulated by the arousal state^4–6^. Histamine H_1_ and H_2_ receptors (H_1_R and H_2_R, respectively) are expressed postsynaptically in most brain regions, including the cerebral cortex^7^. Histamine induces excitatory effects on cortical neurons, as exemplified by depolarization via a decrease in the leaked K^+^ current^8^, which contributes to various brain functions.

Histamine H_3_ receptor (H_3_R) antagonists/inverse agonists have the potential to treat several neuropsychiatric disorders^9^. H_3_Rs in the axon terminals and somas of histamine neurons negatively regulate histamine release and synthesis^10^. They are constitutively active; thus, their antagonists/inverse agonists enhance histamine release^11^. Histamine is a key mediator of learning and memory^12^; therefore, an H_3_R antagonist/inverse agonist is a promising therapeutic agent for improving cognitive impairment^9,12–15^. H_3_R antagonists/inverse agonists enhance memory consolidation and retrieval^16,17^, besides restoring the retrieval of forgotten memories long after learning and forgetting^18^. In addition, they exert antiepileptic actions by increasing histamine release. They increase gamma-aminobutyric acid (GABA) release through the inhibition of H_3_R heteroreceptors, which leads to an antiepileptic effect. H_3_Rs are abundantly expressed in the brain, and the memory-related and antiepileptic effects of H_3_R antagonists/inverse agonists partially involve the modulation of neuronal activity in the cerebral cortex^18–20^. However, the mechanism underlying the influence of H_3_R antagonists/inverse agonists on cortical neuronal activity in vivo is unknown.

In considering the effects of H_3_R antagonists/inverse agonists, it may be useful to refer to the effects of histamine and H_1_R and H_2_R agonists on cortical neuronal activity. Studies using brain slices have demonstrated that histamine application principally induces excitatory effects in cortical neurons^8^, thereby raising the possibility that H_3_R antagonists/inverse agonists merely upregulate cortical activity in vivo. However, this should not be the case because H_3_R antagonists/inverse agonists exert an antiepileptic effect, which likely contradicts the upregulation of cortical activity, thus suggesting a unique mechanism in vivo. Single-unit recordings in vivo have revealed histamine-sensitive neurons predominantly depressed by histamine^21,22^. Histamine supposedly enhances the activity of some neurons in vivo because of its depolarizing effect in brain slices; nonetheless, its impact on the cortical activity in vivo and at a neuronal population level is unknown. This can be attributed to the limited number of simultaneously recorded neurons in these studies. Taken together, it is essential to understand the effects of H_3_R antagonists/inverse agonists on neuronal activity to record the activity in multiple neurons in vivo.

Herein, we aimed to investigate the mechanism by which pitolisant, an H_3_R antagonist/inverse agonist, influences the activity of perirhinal cortex neurons in freely moving mice. Pitolisant has been approved in the EU and USA for the treatment of narcolepsy^23,24^, and preclinical studies have revealed that it enhances the retrieval of novel object recognition memory^25^, which depends on perirhinal cortex (PRh) activity^26^. We intended to record neuronal activity using in vivo Ca^2+^ imaging from mice in their home cages.

## Results

### Pitolisant markedly alters the activity of a subset of neurons without affecting the mean activity across neurons in the PRh

**Figure 1:**
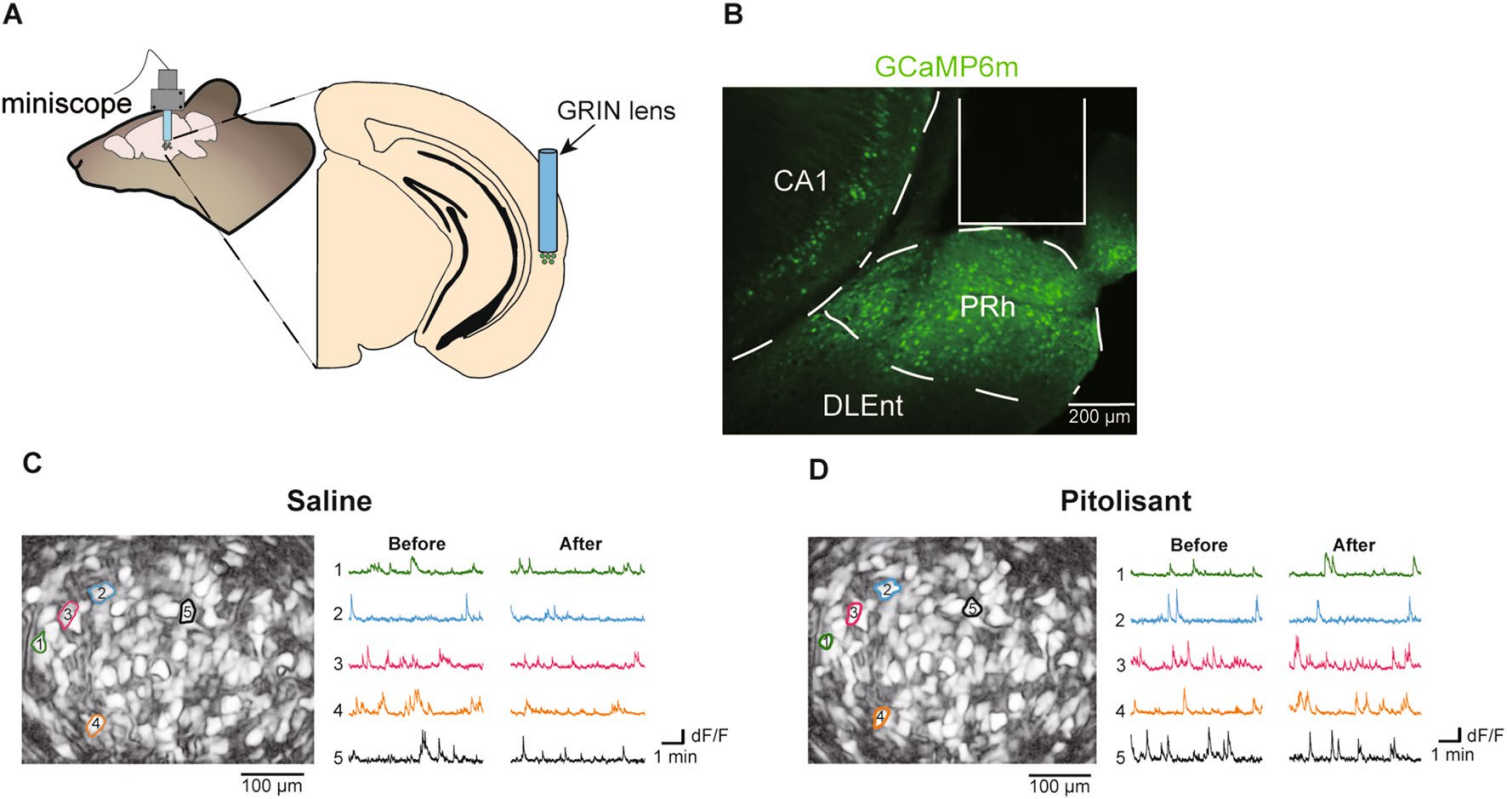
In vivo Ca^2+^ imaging from perirhinal cortex neurons. **(A**) GRIN lens is implanted above the PRh, and images are acquired using the GRIN lens and miniscope. (**B**) A representative image depicting the GRIN lens position and GCaMP6m expression in PRh neurons. (**C**,**D**) Representative correlation images (left) and extracted Ca^2+^ traces before and after saline (**C**) or pitolisant (**D**) injection. DLEnt, dorsolateral entorhinal cortex; GRIN, gradient-index; and PRh, perirhinal cortex.

We recorded neuronal activity in the PRh of freely moving mice using in vivo Ca^2+^ imaging to determine the mechanism underlying pitolisant-mediated alterations in the perirhinal cortex neuronal activity (Fig. 1). We injected a virus into the PRh for delivery of the Ca^**2+**^ sensor protein GCaMP6m under the control of the calcium/calmodulin-dependent protein kinase IIα (CaMKIIα) promoter (AAVdj-CaMKIIα-GCaMP6m), which putatively targets cortical excitatory neurons. Subsequently, we implanted a gradient-index lens above the injection site to obtain fluorescence changes (Fig. 1A,B). Following a minimum of 5 weeks to allow sufficient time for GCaMP6m expression, we visualized GCaMP6m-expressing PRh neurons using a miniaturized one-photon microscope (UCLA Miniscope) from mice that freely moved in their home cage. These mice underwent three imaging sessions (two saline sessions and one pitolisant session in this order) with an interval of at least 24 h. In each session, images were acquired for 10 min, followed by an intraperitoneal injection of saline or pitolisant and image acquisition for 10 min, 30 min later. To determine the effects of pitolisant on the neuronal activity, we compared the data between the pitolisant session and the adjacent saline session. To determine effects of the injection order on the neuronal activity, we compared the data between the first and second saline sessions.

**Figure 2:**
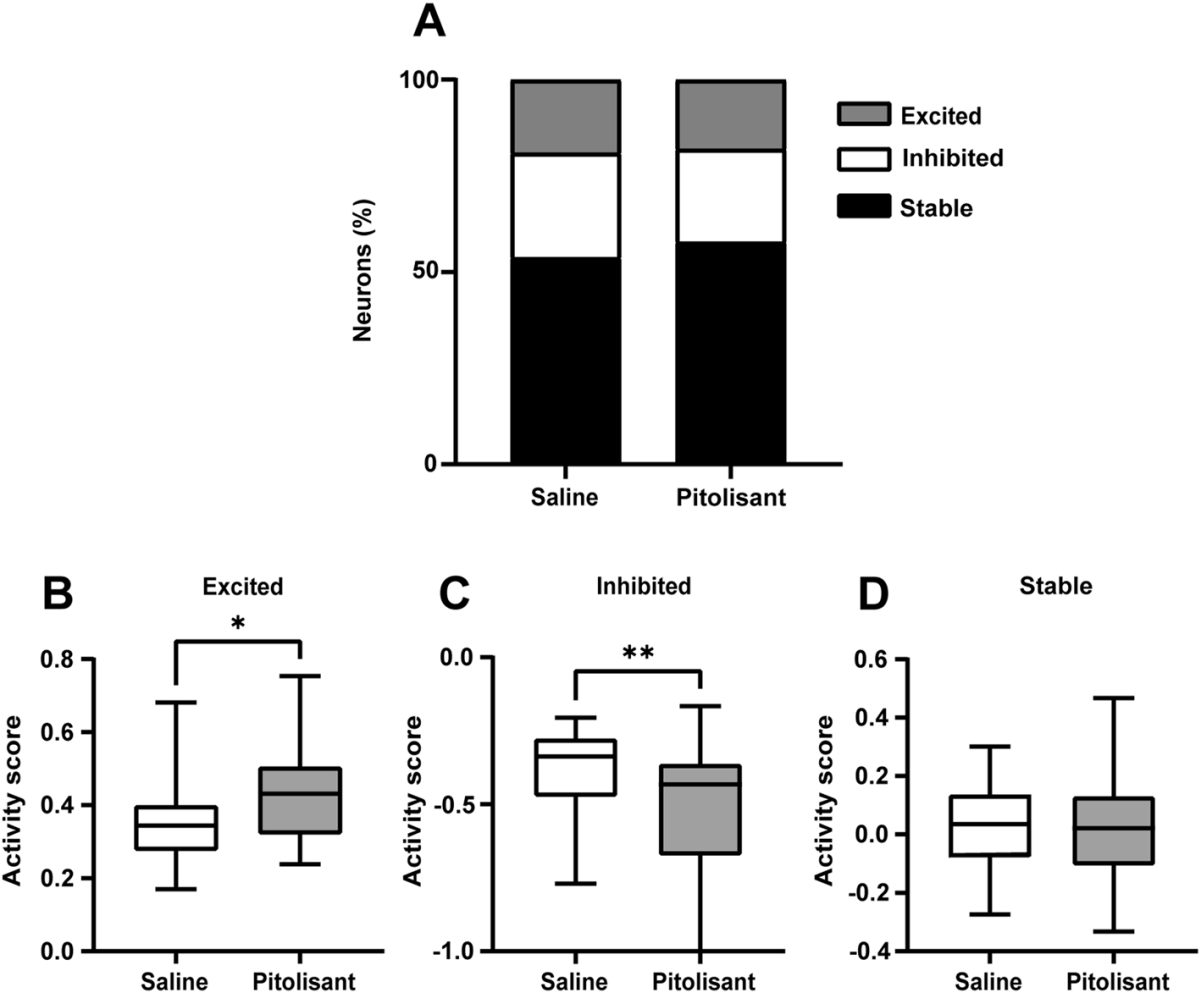
Pitolisant substantially alters the activity of a subset of neurons. (**A**) A proportion of neurons displaying excitatory, inhibitory, or stable responses to saline or pitolisant treatment (saline: 184 neurons, pitolisant: 178 neurons). (**B**) Neurons displaying an excitatory response to pitolisant treatment have higher activity scores, compared with saline treatment (*p = 0.022, Mann–Whitney U test; saline, 35 neurons; pitolisant, 32 neurons). (**C**) Neurons displaying an inhibitory response to pitolisant treatment have lower activity scores, compared with saline treatment (**p = 0.0014, Mann–Whitney U test; saline, 50 neurons; pitolisant, 43 neurons). (**D**) Activity scores of stable neurons are comparable between saline and pitolisant treatments (*p* = 0.5448, Mann–Whitney U test; saline, 99 neurons; pitolisant, 103 neurons).

To evaluate changes in neuronal activity following saline or pitolisant administration, we performed deconvolution of the dF/F signals to infer the spiking activity (Fig. S1) and computed the activity scores of individual neurons. The activity score, an index of neuronal response to saline or pitolisant injection, was calculated as the inferred spiking (deconvolved) activity after an injection minus the activity before the injection, divided by the total activity. It ranges from − 1 (inhibitory response) to + 1 (excitatory response). The mean activity scores across all neurons were comparable between the saline and pitolisant treatments (saline: − 0.020 ± 0.0213, pitolisant: − 0.038 ± 0.0266; *P* = 0.607, Student’s t-test). Consequently, we defined the excitatory and inhibitory responsive neurons to saline and pitolisant administration based on a comparison between their real activity scores and those from their resampled data. The proportion of neurons displaying excitatory and inhibitory responses was comparable between the saline and pitolisant groups (Fig. 2A). The presence of neurons that revealed excitatory and inhibitory responses even after saline treatment may be attributed to changes in the spontaneous activity over time or to the intraperitoneal injection itself. We tracked the same neurons across the two imaging sessions (saline and pitolisant) and found that the excitatory/inhibitory responsive neurons to the pitolisant injection are not associated with those to the saline injection (Supplementary Table 1). Subsequently, we compared the activity scores of excited, inhibited, and stable neurons between treatments. The scores of excitatory responsive neurons to pitolisant were higher than those to saline, whereas the scores of inhibitory responsive neurons to pitolisant were lower than those to saline (Fig. 2B–D). The scores of stable neurons were comparable between saline and pitolisant. For the control experiment, we compared data between two successive saline sessions. The scores of excited, inhibited, and stable neurons were comparable between the first and second saline treatments (excited, *P* = 0.114; inhibited, *P* = 0.590; and stable, *P* = 0.206). Therefore, pitolisant increased the activity of some PRh neurons, while decreasing the activity of others.

### Pitolisant alters the frequency of calcium events from excitatory and inhibitory responsive PRh neurons

**Figure 3:**
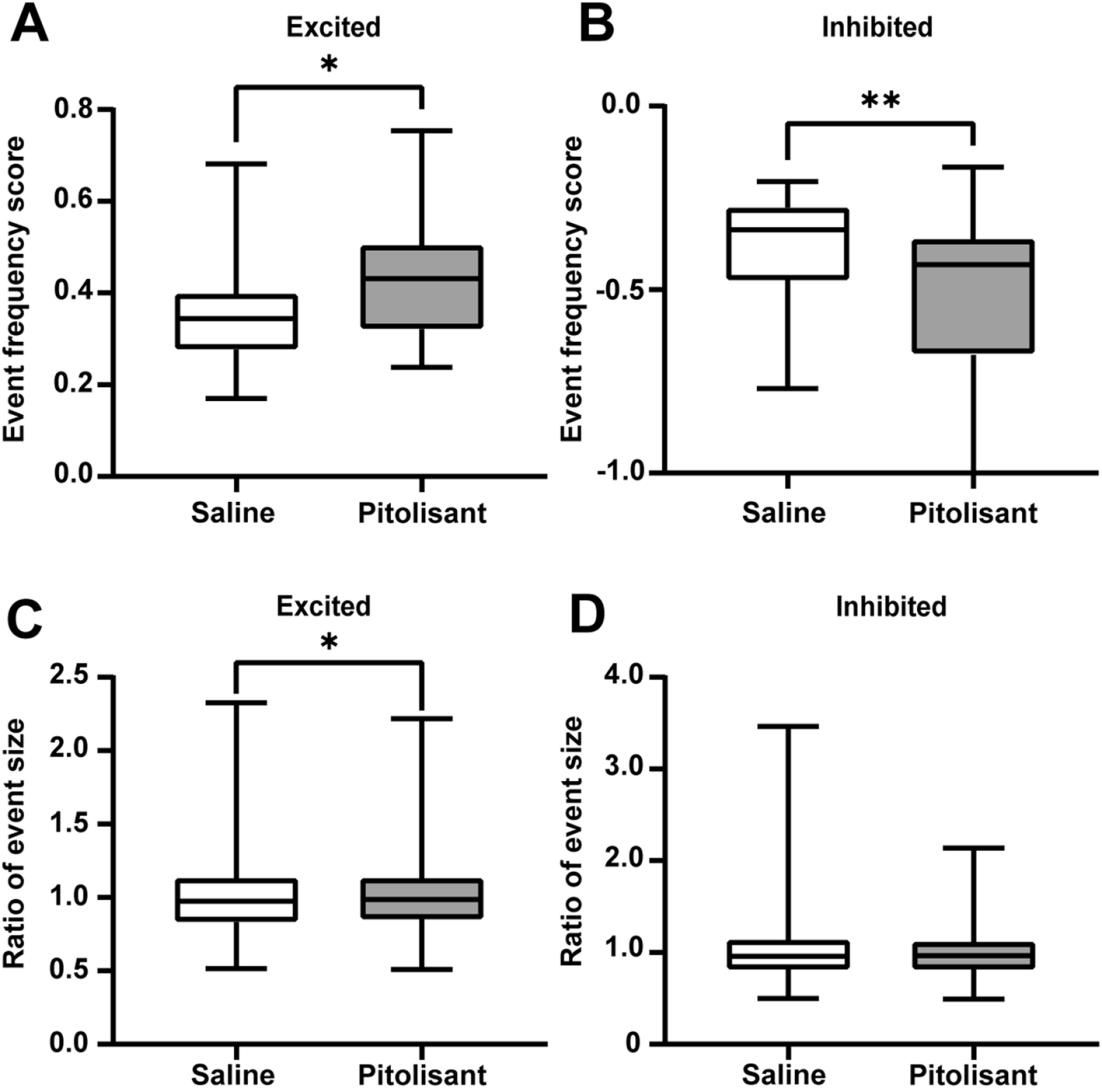
Calcium event frequency from excitatory and inhibitory responsive neurons differ between saline and pitolisant treatments. (**A**) The event frequency score of excitatory responsive neurons to pitolisant treatment is higher compared with saline treatment (*p = 0.029, Mann–Whitney U test). (**B**) The event frequency score of inhibitory responsive neurons to pitolisant treatment is lower compared with saline treatment (**p = 0.0030, Mann–Whitney U test). (**C**) The size of calcium events following pitolisant treatment is larger compared with saline treatment (*p = 0.021, Mann–Whitney U test). (**D**) The size of calcium events from inhibitory responsive neurons is comparable between saline and pitolisant treatments.

To determine factors responsible for changes in activity scores, we compared the frequency and size of calcium events between saline and pitolisant. Excitatory responsive neurons to pitolisant displayed a higher frequency of calcium events than that to saline, whereas inhibitory responsive neurons to pitolisant displayed a lower frequency than that to saline (Fig. 3A,B). While the calcium event size of excitatory responsive neurons to pitolisant was moderately larger than that to saline, there was no difference between the calcium event sizes of inhibitory responsive neurons to pitolisant and saline (Fig. 3C,D). In other words, the increase and decrease in neuronal activity following pitolisant administration were primarily because of changes in the frequency of calcium events.

### Pitolisant increases neuron pairs with synchronous activity within the excitatory responsive neuronal populations

**Figure 4:**
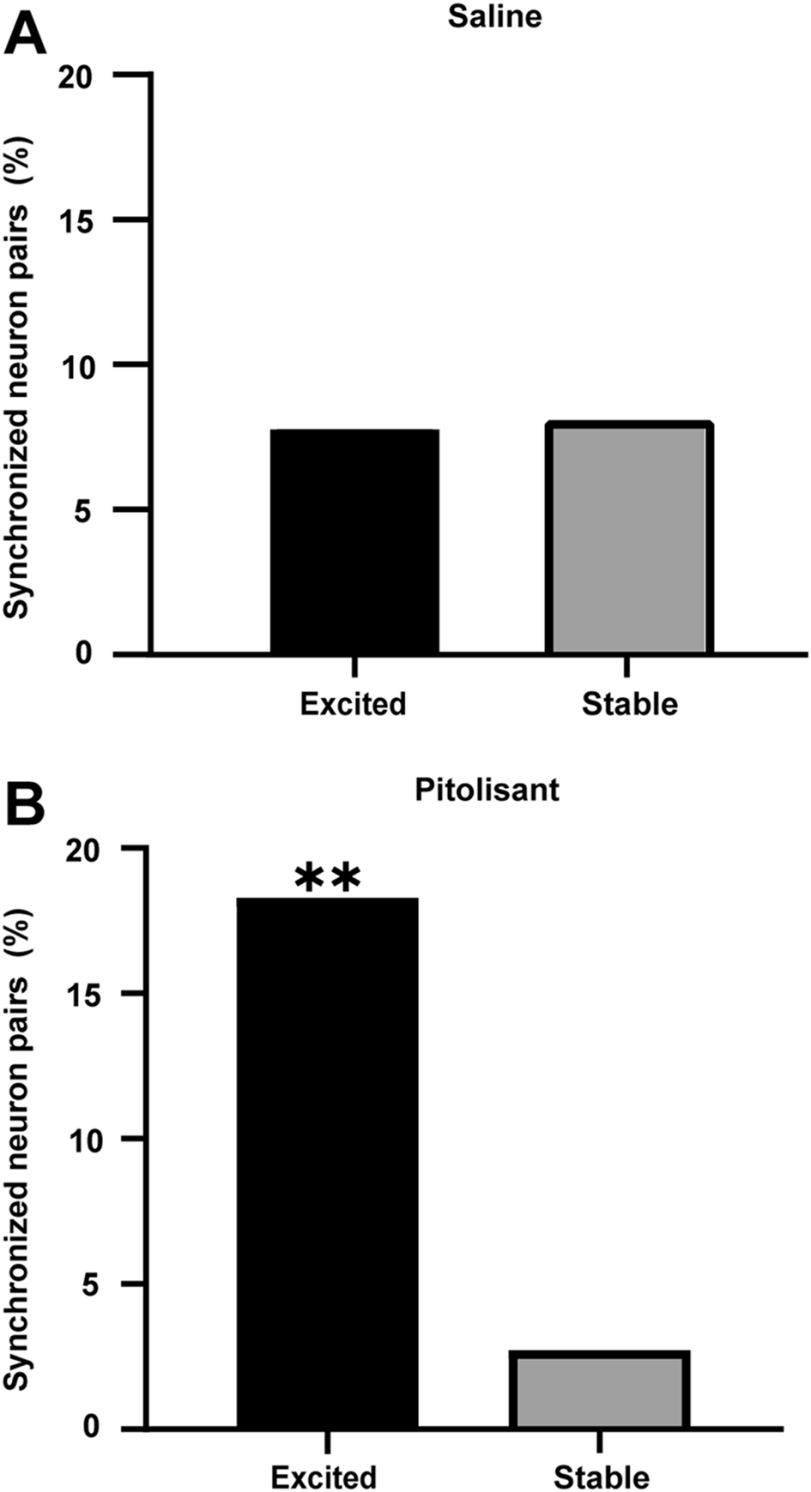
Pitolisant enhances the proportion of synchronously active neuron pairs within excitatory responsive neuronal populations. (**A**) Proportions of cell pairs with synchronous activity do not differ between neuronal populations displaying excitatory and stable responses to saline treatment (*p* = 0.81, Fisher's exact test). (**B**) More cell pairs display synchronous activity within excitatory responsive neuronal populations to pitolisant treatment, compared with stable neuronal populations (**p = 6.2 × 10^–5^, Fisher's exact test).

Neuronal synchronization is important for carrying information, perception^27^, memory formation^28^, and memory retrieval^29^. Thus, we examined the effect of pitolisant on synchronous activity. To evaluate the synchronicity of the two neurons, we computed the cross-correlogram (CCG), which was computed using the deconvolved activity trains of the two neurons and was normalized using the total activity of the two neurons. A synchronous neuron pair was defined based on the comparison between the real CCG and one computed from temporally shuffled data. Following saline treatment, the proportion of cell pairs with synchronous activity did not differ between excited and stable neuronal populations (Fig. 4A). In contrast, following pitolisant treatment, more cell pairs revealed synchronous activity within the excited neuronal populations than those in stable neuronal populations (Fig. 4B).

### Pitolisant alters PRh neuronal population activity

**Figure 5:**
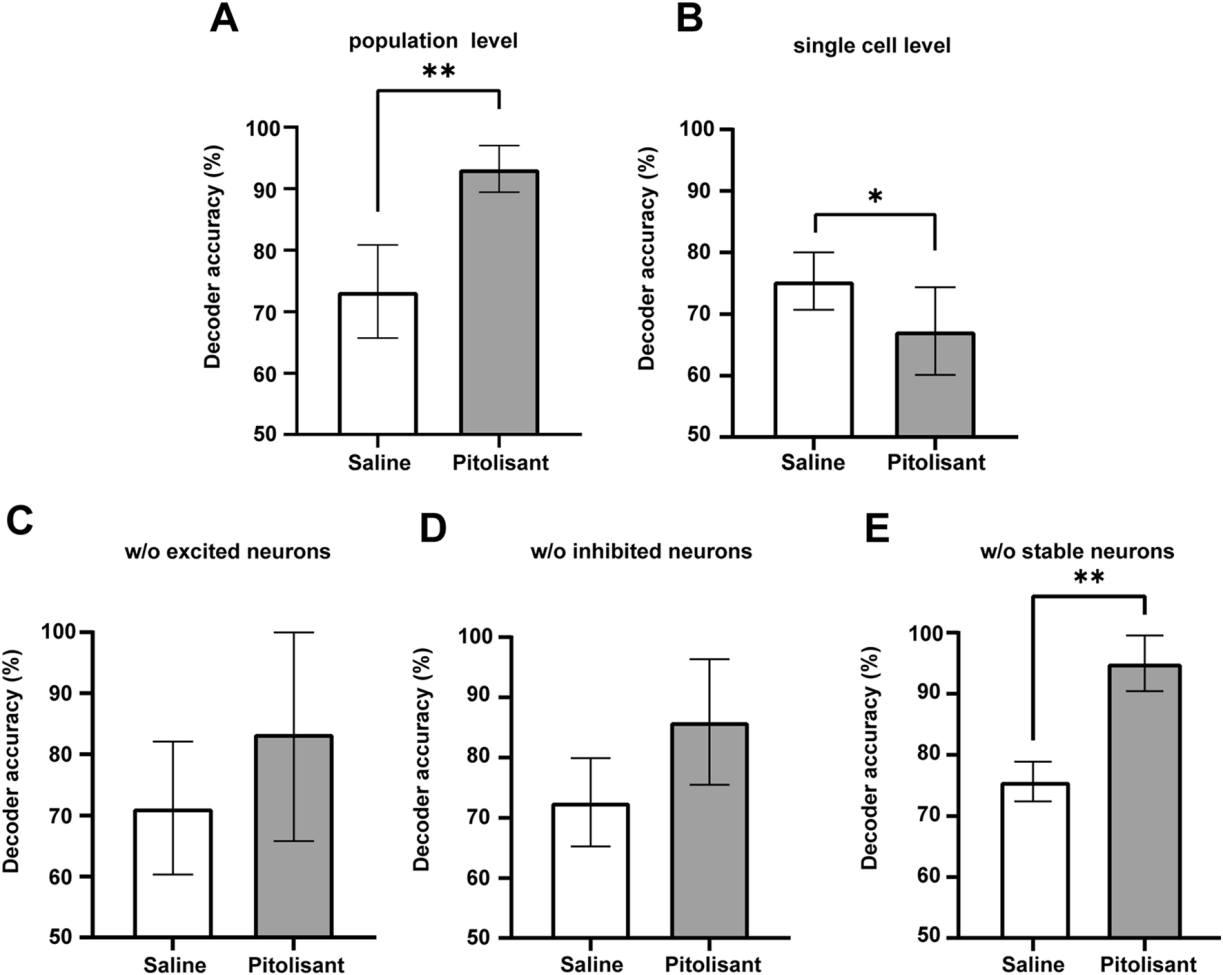
Population activity before and after pitolisant treatment is accurately classified by linear support vector machine. (**A**) Accuracy of two-way decoders discriminating before and after treatment. Decoders based on population activity data before and after pitolisant treatment performed better, compared with saline treatment (**p = 0.0053, paired t-test). (**B**) Decoders constructed from single neuron data before and after pitolisant treatment performed poorer, compared with saline treatment (*p = 0.045, paired t-test). (**C**) Decoders constructed from population activity data without excitatory responsive neurons performed similarly between saline and pitolisant treatments (*p* = 0.3020, paired t-test). (**D**) Decoders constructed from population activity data without inhibitory responsive neurons performed similarly between saline and pitolisant treatments (*p* = 0.0897, paired t-test). (**E**) Decoders constructed from population activity data without stable neurons performed better than those treated with saline (**p = 0.0038, paired t-test). Data are reported as mean ± SEM.

To determine the impact of pitolisant on neuronal population activity, we compared the performance of the decoder, which distinguished imaging epochs (before or after treatment) between saline and pitolisant based on the neuronal population activity. Decoder accuracy was evaluated using cross-validation, in which the data were divided into a ratio of 7:3. The decoder distinguished the neuronal population activity before and after pitolisant treatment with a 93.1 ± 1.5% accuracy rate, thereby indicating pitolisant substantially altered the neuronal population activity. This decoder accuracy was higher than that based on saline data (73.8 ± 0.030%) (Fig. 5A). We also examined the decoder accuracy of the single neurons. The mean accuracy of the decoders discriminating before and after the pitolisant treatment from single neurons (67.2 ± 3.2%) was slightly lower than that based on the saline data (75.3 ± 2.1%) (Fig. 5B). This suggests that the high accuracy of decoders constructed from the population data is not just reflective of performance from the single neurons.

To determine the contribution of excited, inhibited, and stable neurons to decoder accuracy, we constructed decoders based on the data that excluded these neurons and compared the decoder performance between treatments. The decoders constructed without excited or inhibited neurons performed similarly between saline and pitolisant treatments (Fig. 5C,D). By contrast, the decoder based on pitolisant data, which excluded stable neurons, performed better than that based on saline data (Fig. 5E). Thus, pitolisant altered neuronal population activity, which depended on excitatory-and inhibitory-responsive neurons.

## Discussion

In this study, we monitored neuronal activity in freely moving mice and demonstrated that pitolisant influenced the PRh activity in individual neurons and neuronal population levels in vivo. Pitolisant increased the activity of some PRh neurons, while decreasing the activity of others. Pitolisant did not affect the mean activity across neurons, possibly because the increase and decrease offset each other. The change in the activity of excitatory-and inhibitory-responsive neurons to pitolisant was primarily because of a change in the frequency of calcium events. Pitolisant increased synchronously active neuron pairs in excitatory responsive neuronal populations and altered neuronal population activity, which was dependent on excitatory and inhibitory neurons responsive to pitolisant treatment. In other words, pitolisant influenced the activity of a subset of PRh neurons by increasing the synchronous activity and modifying the ensemble representation, without affecting the mean activity across neurons in the PRh.

Effects of pitolisant on neuronal activity may be attributed to the histamine-induced excitatory effects. The dose of pitolisant used in this study (20 mg/kg) presumably enhanced histamine release in the PRh. This is because a smaller dose (10 mg/kg) increases brain *tele*-methylhistamine levels, an index of histaminergic neuron activity^25^, and thioperamide, another H_3_ receptor antagonist, enhances histamine release in the PRh^18^. Previous experiments using brain slices revealed that histamine H_1_R and H_2_R activation exert an excitatory effect on neurons in most brain regions, including the PRh^8,18,30^. Therefore, pitolisant may induce histamine release in the PRh, which in turn triggers the excitatory effect on PRh neurons. However, pitolisant did not affect the mean activity from the recorded PRh neurons, and some PRh neurons displayed an inhibitory response, which could be explained by the homeostatic regulation of cortical activity. Excitatory and inhibitory synaptic inputs are balanced in healthy neural circuits. Moreover, excitability and spontaneous firing frequency are maintained within a certain range, possibly owing to dense local connectivity^31^. Previous single-unit recording studies have demonstrated that histamine suppresses the activity of some neurons in an H_2_R-dependent manner^21,22^. This inhibitory effect was blocked by a GABA_A_ receptor antagonist, thereby suggesting histamine suppresses cortical neurons by activating inhibitory interneurons. Another possible mechanism underlying the effects of pitolisant is the modulation of other neurotransmitters through the activation of heteroreceptors^32^. H_3_ receptors regulate histamine release as well as several other neurotransmitters (e.g., dopamine, GABA, acetylcholine, and noradrenaline). Thioperamide increases GABA release in the cortex^33^. Pitolisant may induce GABA release in the PRh, thus explaining the inhibitory response of some neurons to pitolisant treatment. It is important to note that the experimental approaches are not completely aligned between previous studies showing the excitatory effect of histamine and the current study as the previous studies examined the effects of histamine ex vivo and the current study examined the effects of pitolisant in vivo. These warrant future analysis using injection of histamine, co-injection of antagonists, including H_1_, H_2_, and other receptor antagonists, and/or the genetic or chemogenetic manipulation of histamine neurons^34,35^ to determine the mechanisms underlying pitolisant-mediated effects in vivo.

The selection of neurons for excitatory or inhibitory responses to pitolisant warrants investigation. Different expression levels of histamine H_1_ and H_2_ receptors in neurons may affect the responsiveness to pitolisant. The activity history of individual neurons is another possible factor that determines responsiveness to pitolisant. In our previous study, we compared neuronal excitability in brain slices between activated and inactivated neurons during the novel object recognition task. In the absence of histamine, the responsiveness to electrical stimulation was comparable; however, neurons that were activated during learning were preferentially activated by the stimulation in the presence of histamine^18^. A plastic change followed by prior behavioral experience is likely to affect the effects of pitolisant.

The excitatory response of some neurons to pitolisant may contribute to their ability to promote memory retrieval. H_3_ receptor antagonists/inverse agonists, including pitolisant, enhance memory retrieval, which is mediated by the specific reactivation of neuronal subpopulations activated during learning^36,37^. For example, a neuronal subpopulation in the basolateral amygdala active during fear conditioning gets reactivated during the subsequent retrieval of fear memory^38–40^. Additionally, the optogenetic activation of dentate gyrus neurons that were activated during fear learning results in the retrieval of fear memory^41^. In particular, a population of ventral CA1 neurons, including neurons activated during learning, display synchronized activity that is proportional to the strength of contextual fear memory retrieval^29^. Histamine preferentially reactivates PRh neurons activated during object learning in brain slices^18^. Therefore, pitolisant treatment followed by learning may upregulate the synchronized reactivation of PRh neurons activated during learning by increasing histamine release, which promotes memory retrieval. However, the mechanism underlying the storage and retrieval of object memories in PRh neurons is unclear^42–45^. This is because most studies on memory engram cells have targeted the hippocampus and amygdala. Therefore, future studies using in vivo recordings during behavioral tasks and the selective activity manipulation of excitatory responsive neurons will provide insights into the mechanisms by which pitolisant promotes memory retrieval.

In conclusion, pitolisant substantially upregulated and downregulated the activity of a subset of PRh neurons, bedsides increasing synchronous activity within the excitatory responsive neuronal populations and modifying the ensemble representation. A CCG analysis revealed that pitolisant increased synchronously active neuron pairs within excitatory-responsive neuronal populations. Machine learning analysis revealed that it altered the neuronal ensemble activity, which depended on excitatory and inhibitory responsive neurons. Collectively, our findings provide essential knowledge on the process by which pitolisant modulates cortical neuronal activity and brain function. These modulatory effects may contribute to the promotion of memory retrieval. Further investigation into the impact of pitolisant and other H_3_ receptor antagonists/inverse agonists on neuronal activity during memory tasks will shed light on the mechanism underlying enhanced learning and memory.

## Methods

### Animals

Animal experiments were performed with the approval of the Animal Experiment Ethics Committee at the Hokkaido University (approval number: 16-0043) and according to the Hokkaido University Guidelines for the Care and Use of Laboratory Animals. These experimental protocols were conducted in accordance with the Fundamental Guidelines for Proper Conduct of Animal Experiments and Related Activities in Academic Research Institutions (Ministry of Education, Culture, Sports, Science and Technology, Notice No. 71 of 2006), the Standards for Breeding and Housing of and Pain Alleviation for Experimental Animals (Ministry of the Environment, Notice No. 88 of 2006), and the Guidelines on the Method of Animal Disposal (Prime Minister's Office, Notice No. 40 of 1995). Animal use follows the recommendations of the ARRIVE guidelines^46^.

Five adult male C57BL/6 J mice (8–20 weeks old; Japan SLC, Hamamatsu, Japan) were used in this study. They were housed in a room with a constant ambient temperature (23 ± 1 °C) under a 12-h light/dark cycle (lights on from 7 a.m. to 7 p.m.), with food and water available ad libitum.

### Drugs

Pitolisant maleate (BF2.649) (Tocris Bioscience, Bristol, United Kingdom) was dissolved in an isotonic saline solution (0.9% NaCl) and administered at a volume of 0.01 ml/g body weight via the intraperitoneal (i.p.) route. The control treatment consisted of an equal volume of saline. The pitolisant dose (20 mg/kg) was selected based on previous studies^47,48^.

### Surgery

The mice were intraperitoneally injected with carprofen (5 mg/kg) and dexamethasone (0.2 mg/kg), anesthetized with isoflurane (0.8–1.5%), and placed in a stereotaxic frame (SR-6M-HT, Narishige, Tokyo, Japan). Lidocaine (2%; Aspen Japan, Tokyo, Japan) was applied topically to the scalp to alleviate pain. We injected AAVdj-CaMKIIα-GCaMP6m (1.9 × 10^13^ virus molecules/mL, 0.5 μL) into the unilateral PRh (A/P: − 3.0 mm, M/L: ± 4.5 mm, D/V: − 3.95 mm) at a rate of 0.1 μL/min. The infusion cannulas (33 gauge) were left in place for at least 10 min to facilitate the diffusion of the solutions. We implanted a Gradient Index (GRIN) lens (0.6 mm in diameter, 7.3 mm in length; Inscopix, Palo Alto, CA) 0.1 mm dorsal to the PRh target site and used two screws to anchor it to the skull. The GRIN lens and screws were fixed with a self-curing adhesive resin cement (Super-Bond, SUN MEDICAL, Moriyama, Japan). A Kwik-Sil (World Precision Instruments) was used to cover the GRIN lens. Carprofen, dexamethasone, and amoxicillin (50 mg/kg) were administered for 7 days post-surgery.

The mice underwent baseplate surgery ≥ 5 weeks following the initial surgery. The mice were anesthetized with isoflurane and placed in a stereotaxic frame. After removing the Kwik-Sil, a miniscope (UCLA miniscope V3.2, LABMAKER, Berlin, Germany; UCLA miniscope V4, Open Ephys, Lisbon, Portugal) with a baseplate was positioned such that the field of focus was in view. The baseplate was secured to the previously formed cement using additional cement. After detaching the miniscope, a plastic cover was placed on the base plate to protect the GRIN lens.

### In vivo Ca^2+^ imaging

Prior to imaging, the mice underwent habituation sessions over 2 days. In each habituation session, they were briefly anesthetized with isoflurane, and a dummy miniscope was attached to the baseplate. They were returned to their home cage that was placed in a sound attenuating chamber. They received an intraperitoneal injection of saline 40 min later and stayed in the home cage for additional 40 min. After the next day, the mice underwent three imaging sessions (two saline sessions and one pitolisant session in this order) at intervals of at least 24 h. In each imaging session, they were briefly anesthetized with isoflurane, a miniscope was attached to the baseplate, and they were returned to their home cage that was placed in the sound attenuating chamber. They were allowed to recover from anesthesia for 30 min prior to the beginning of imaging. Images were acquired with a miniscope and data acquisition hardware for 10 min, followed by saline or pitolisant injection and image acquisition for 10 min, 30 min later.

### Data analysis

We performed calcium imaging analysis using CaImAn^49^. The obtained raw images were converted into 8-bit images using Fiji (National Institutes of Health, https://fiji.sc/) and motion corrected. The regions of interest (ROIs) and time series of their fluorescence signals were generated using constrained non-negative matrix factorization^50^. The automatically extracted ROIs were manually inspected for quality control. Across the individual ROIs, we calculated the dF/F as the fluorescence change divided by the baseline fluorescence. The dF/F traces were deconvolved to estimate neuronal activity (spiking activity). The deconvolved activity (arrays of estimates.S generated by CaImAn) was approximately proportional to the firing rate each time. To track the same neurons across two imaging sessions, the centroid distances were measured from each cell in the saline session to the most proximate cell acquired from the pitolisant session, and pairs of neurons with a distance shorter than 6.5 μm were considered the same neurons.

To evaluate changes in neuronal activity following drug administration, we calculated the activity scores of individual neurons using the following formula:

$$Activity\;score = \frac{{A_{after} - A_{before} }}{{A_{after} + A_{before} }}$$

where $A_{before}$ and $A_{after}$ denote the accumulation of deconvolved neuronal activity over a recording session before and after administration, respectively.

We excluded neurons that were highly active or extremely silent before drug administration because we cannot determine effects of the drug administration on these neurons. The lower limit of A_before_ was set at 1000 (a.u.) because the activity score of the neurons whose A_before_ was lower than this threshold was higher than zero (0.63 ± 0.051, *p* < 0.0001, one sample t-test) even after saline treatment. The upper limit of A_before_ was set at 4000 (a.u.) because the activity score of the neurons whose A_before_ was higher than this threshold was lower than zero (− 0.26 ± 0.019, *p* < 0.0001, one sample t-test) even after saline treatment.

We temporally shuffled the deconvolved activity data and obtained a distribution of the activity scores based on the shuffled data (100,000 iterations). Neurons with real scores higher than the top 0.1% and lower than the bottom 0.1% of the scores from shuffled data were defined as excitatory and inhibitory responsive neurons to drug administration.

Calcium events were defined as a deconvolved neuronal activity with an amplitude > 0. To assess the effects of drug treatment on the size of calcium events, we calculated the normalized amplitude of each event as the ratio of the amplitude of the event from a neuron to the mean event amplitude before the treatment from the same neuron.

Calcium event frequency was calculated based on the number of calcium events recorded in a 10-min period. To analyze changes in calcium event frequency following drug administration, the frequency scores of individual neurons were computed as follows:

$$Frequency\;score = \frac{{F_{after} - F_{before} }}{{F_{after} + F_{before} }}$$

where $F_{before}$ and $F_{after}$ denote the frequency of calcium events before and after administration, respectively.

To assess the synchronicity of a neuron pair, the cross-correlogram (CCG)^51^ was computed as follows:

$$CCG = max \frac{{\mathop \sum \nolimits_{t = 1}^{T} x_{1} (t)x_{2} (t + \tau )}}{{\sqrt {s_{1} * s_{2} } }}$$

where $T$ is the number of imaging frames, $x_{1}$ and $x_{2}$ are the deconvolved activity trains of the two neurons, $s_{1}$ and $s_{2}$ are their total activities, and $\tau$ is the time lag (− 1, 0, or + 1). We temporally shuffled the deconvolved activity data and obtained a CCG distribution based on the shuffled data (10,000 iterations). Neuron pairs with real CCGs higher than the top 1% of CCGs from the shuffled data were defined as synchronous pairs. We used CCG normalized by the activity of the two neurons; nonetheless, low activity affected the proportion of the synchronous pairs. For example, stable neurons with lower activity ($A_{after}$ < 3000) were less judged to be synchronously paired (0.64%) than those with higher activity ($A_{after}$ ≥ 3000) (2.5%) following saline treatment (*P* = 3.6 × 10^–4^, Fisher's exact test). Therefore, neurons with low activity ($A_{after}$ < 3000) were excluded from the analysis of synchronicity. We calculated the proportion of neuron pairs with synchronous activity within excitatory- and non-responsive neuron populations to drug treatment. We did not analyze the synchronicity within inhibited neuron populations because all of the inhibited neurons were excluded from the criterion.

We examined the impact of drug treatment on the neuronal ensemble activity by using the Scikit-learn package as follows. We down-sampled the 20-min deconvolved neuronal activity data (a matrix of the number of cells × 12,000 frames) into 30-s time bins (a matrix of the number of cells × 40 bins). To avoid the effects of dataset size between the treatments, a random subset of neurons was selected from the data with a higher number of neurons such that the number of neurons was aligned within the mouse. We used the aforementioned activity data with the imaging epoch labels (an array of 40 labels (before or after treatment)) to train a linear support vector machine decoder. The decoder performance was evaluated using cross-validation by dividing the data into 70% and 30% for training and testing, respectively. The model was regularized with an L1 penalty to prevent overfitting. To evaluate the decoder performance from the data with a higher number of neurons within a mouse, the random cell choice and evaluation of the performance were repeated 100 times. Moreover, we computed the decoder accuracy as the average accuracy across 100 randomly selected datasets. To determine if excitatory-, inhibitory-, and non-responsive neurons to a drug treatment contributed to decoder accuracy, we constructed decoders based on the data that excluded these neurons and compared the decoder performance between treatments.

We assessed the decoder accuracy of single neurons in a similar manner to the decoder constructed from neuronal population data. We used deconvolved, down-sampled activity data of individual neurons with the imaging epoch labels to train a linear support vector machine decoder. The mean decoder accuracy across neurons was calculated per mouse and compared between saline and pitolisant treatments.

### Histology

Following the imaging experiments, mice were deeply anesthetized with pentobarbital and transcardially perfused with phosphate buffered saline (PBS), followed by 4% paraformaldehyde (PFA). Brains were post-fixed in 4% PFA overnight at 4 °C, followed by cryoprotection for 48–72 h in 15% and 30% sucrose dissolved in PBS at 4 °C before freezing on dry ice. Coronal slices (40 μm thick) were obtained using a cryostat (CM3050S, Leica). Images were acquired using a fluorescence microscope (10× magnification; BZ-X700, Keyence) to confirm the position of the lens implantation and GCaMP6m expression.

## Supplementary Information

Supplementary Information 1.

Supplementary Information 2.

## Data Availability

All data generated during this study are included in this published article and its supplementary information files.
