# Supplementary material for: The impact of pitolisant, an H3 receptor antagonist/inverse agonist, on perirhinal cortex activity in individual neuron and neuronal population levels
Source: Sci Rep. 2022 May 12;12:7015. doi: 10.1038/s41598-022-11032-y (PMC9098477; doi:10.1038/s41598-022-11032-y)
Supplement: Supplementary file 2 — Supplementary Information 2. [file 41598_2022_11032_MOESM2_ESM.docx]

**Supplementary information**





**Figure S1.**

Representative traces showing that a raw dF/F signal was deconvolved to estimate a spiking activity by using CaImAn.

| Number of neurons | | **Saline** | |
| --- | --- | --- | --- |
|  |  | Excited | Others |
| **Pitolisant** | Excited | 0 | 7 |
|  | Others | 5 | 35 |
|  | | | |
| Number of neurons | | **Saline** | |
|  |  | Inhibited | Others |
| **Pitolisant** | Inhibited | 4 | 7 |
|  | Others | 10 | 26 |

**Supplementary Table 1.**

Relationships between saline-responsive and pitolisant-responsive neurons. The same neurons were tracked across the two imaging sessions. The excited and inhibited neurons by the pitolisant injection were not associated with those by the saline injection (Excited, P > 0.999; Inhibited, P = 0.710; Fisher’s exact test).
